# Nonpharmacological interventions for cancer-related fatigue in lung cancer patients: A protocol for an evidence map of overview of a network meta-analysis of existing trials

**DOI:** 10.1097/MD.0000000000026864

**Published:** 2021-08-13

**Authors:** Lingyan Zhao, Ping Shi, Xiaomin Xiong, Jia Zeng

**Affiliations:** Respiratory and Critical Care Medicinen, Guangyuan Central Hospital, Guangyuan, Sichuan province, China.

**Keywords:** cancer-related fatigue, lung cancer, meta-analysis, nonpharmacological intervention, protocol

## Abstract

**Background:**

Lung cancer is one of the most common cancers, the symptoms and treatment of which can cause negative emotions like anxiety, depression, and cancer-related fatigue (CRF). Nonpharmacological interventions, serving as alternative therapies, can greatly alleviate CRF in lung cancer patients. Previous meta-analyses have reported nonpharmacological interventions of CRF in lung cancer patients, but the results may be conflicting, and the reporting and methodological qualities remain unknown. Moreover, there is limited evidence to identify efficient and safe non-pharmacological interventions of CRF in lung cancer patients. This study aims to assess the therapeutic efficacy of nonpharmacological interventions of CRF in lung cancer patients through a network meta-analysis.

**Methods:**

Relevant literatures reporting non-pharmacological interventions of CRF in lung cancer patients published before June 2021 will be searched in online databases, including Wanfang, VP Information Chinese Journal Service Platform, China National Knowledge Infrastructure, Chinese BioMedicine Literature Database, PubMed, Embase, Cochrane, and Web of science. Two reviewers will be independently responsible for study selection, quality appraisal, and data extraction. Data analysis will be performed using the STATA14.0 and GEMTC 0.14.3 software.

**Results:**

This meta-analysis will provide additional and stronger evidences for nonpharmacological interventions of CRF in lung cancer patients. Our findings will be conductive to make therapeutic decisions by clinicians.

**Conclusion:**

This study will provide a reliable evidence-based basis for non-pharmacological interventions of CRF in lung cancer patients.

**Ethics and dissemination:**

Ethical approval was not required for this study. The systematic review will be published in a peer-reviewed journal, presented at conferences, and shared on social media platforms. This review would be disseminated in a peer-reviewed journal or conference presentations.

**OSF REGISTRATION NUMBER:**

DOI 10.17605/OSF.IO/QRY42.

## 1 Introduction

The latest cancer statistics have shown that there were about 18.1 million new cancer cases and 9.6 million cancer deaths worldwide in 2018.^[1]^ Globally, the top 5 cancers with the highest incidence include the lung cancer, breast cancer, colorectal cancer, prostate cancer, and stomach cancer. The incidence of lung cancer in China remains the highest.^[1]^ With the emergence and application of novel therapeutic strategies for lung cancer like targeted therapy, immunotherapy, and biotherapy, the overall 5-year survival of lung cancer is on the rise. At present, lung cancer treatment is no longer satisfied in the cancer lesion itself, and the alleviation of clinical symptoms and improvement of quality of life in lung cancer patients have been highlighted.^[2]^

Cancer-related pain and vomiting are now effectively controlled. Cancer-related fatigue (CRF), however, is an important factor affecting the quality of life of cancer patients and of great concern to the medical community.^[3]^ CRF refers to a generalized, persistent, subjective feeling of fatigue due to cancer disease or cancer treatment that lasts for months, or even years and cannot be relieved by sleep or rest.^[4,5]^ It is reported that 50% to 90% of cancer patients experience fatigue.^[6]^ However, the incidence of CRF in lung cancer is up to 96%.^[7]^ Persistent CRF not only negatively influences the body and mind, but also affects physiological and psychological functions, social activities and daily life of cancer patients, which seriously reduces the quality of life.^[8–10]^

There is presently no criterion standard treatment for CRF. Nonpharmacological interventions may be effective to lung cancer patients with CRF, including positive meditation, muscle relaxation, yoga, Tai Chi, cognitive behavioral therapy, and acupuncture.^[11–16]^ To date, the effects of different nonpharmacological interventions on CRF in lung cancer patients are inconclusive, and few studies have compared their efficacies.

A network meta-analysis is a tool for comparing and pooling evidences from multiple interventions, which provides a relative ranking of clinical outcomes achieved by these interventions.^[17,18]^ Although many meta-analyses of non-pharmacological interventions of CRF in lung cancer patients have been published,^[19–21]^ their results may be inconsistent or even contradictory. In addition, the reporting and methodological qualities of these meta-analyses are unknown, which may affect the clinical utility and scientific reliability of the results. Therefore, we designed an overview to assess the reporting and methodological qualities of meta-analyses of non-pharmacological interventions of CRF in lung cancer patients. In addition, a network meta-analysis will be conducted to compare the relative effectiveness and safety of nonpharmacological interventions reported in the randomized controlled trials (RCTs) involving in this overview of meta-analyses for CRF in lung cancer patients.

## 2 Methods

### 2.1 Study registration

The protocol of this review will be registered in OSF Registries (OSF registration number: DOI 10.17605/OSF.IO/QRY42), which follows the statement guidelines of preferred reporting items for systematic reviews and meta-analyses protocol.^[22]^

### 2.2 Inclusion criteria for study selection

1. Study type: RCTs, systematic reviews, and meta-analyses.
2. Participants: Patients who are pathologically diagnosed as lung cancer. The nationality, race, sex, and age of the patients included in the study will not be restricted. No restriction will be made on the tumor staging and pathological subtype of lung cancer.
3. Interventions: Lung cancer patients in the intervention group are managed by nonpharmacological intervention programs, such as aerobic exercise, acupuncture, yoga, massage, and so on, whereas those in the control group are intervened by conventional treatments.
4. Outcome indicators: Any rating scales that describe CRF, anxiety, and depression.

### 2.3 Exclusion criteria

1. Duplicate publications.
2. Incomplete data.
3. Studies with inconsistent outcomes.

### 2.4 Data sources

Wanfang, VP Information Chinese Journal Service Platform, China National Knowledge Infrastructure, Chinese BioMedicine Literature Database, PubMed, Embase, Cochrane, and Web of Science will be systematically searched. In addition, citations in the included systematic reviews of meta-analyses will be examined to prevent missing data. The time for literature retrieval will be set from the establishment of the database until June 2021.

### 2.5 Searching strategy

A combination of subject terms and free words will be adopted in the searching strategy. Searching strategies using the PubMed were illustrated in Table 1, and literature search in other online databases will be similarly conducted.

**Table 1:** Search strategy in PubMed database.

Number	Search terms
#1	Lung Neoplasms[MeSH]
#2	Cancer of Lung[Title/Abstract]
#3	Lung Cancer[Title/Abstract]
#4	Pulmonary Cancer[Title/Abstract]
#5	Pulmonary Neoplasms[Title/Abstract]
#6	Cancer of the Lung[Title/Abstract]
#7	Neoplasms, Lung[Title/Abstract]
#8	Neoplasms, Pulmonary[Title/Abstract]
#9	Cancer, Lung[Title/Abstract]
#10	Cancer, Pulmonary[Title/Abstract]
#11	Cancers, Lung[Title/Abstract]
#12	Cancers, Pulmonary[Title/Abstract]
#13	Lung Cancers[Title/Abstract]
#14	Lung Neoplasm[Title/Abstract]
#15	Neoplasm, Lung[Title/Abstract]
#16	Neoplasm, Pulmonary[Title/Abstract]
#17	Pulmonary Cancers[Title/Abstract]
#18	Pulmonary Neoplasm[Title/Abstract]
#19	OR/1–18
#20	Cancer-related fatigue[Title/Abstract]
#21	CRF [Title/Abstract]
#22	OR/20–21
#23	Systematic Review [Publication Type]
#24	Systematic Reviews as topic[MeSH]
#25	Network Meta-Analysis[MeSH]
#26	Meta-analysis [Publication Type]
#27	Meta-analysis as topic[MeSH]
#28	Systematic review[Title/Abstract]
#29	Meta-analysis[Title/Abstract]
#30	Randomized Controlled Trials as Topic[MeSH]
#31	Clinical Trials, Randomized[Title/Abstract]
#32	Controlled Clinical Trials, Randomized[Title/Abstract]
#33	Trials, Randomized Clinical[Title/Abstract]
#34	Random∗[Title/Abstract]
#35	OR/23–34
#36	#19 AND #22 AND #35

### 2.6 Data collection and analysis

#### 2.6.1 Literature screening and data extraction

**Figure 1:**
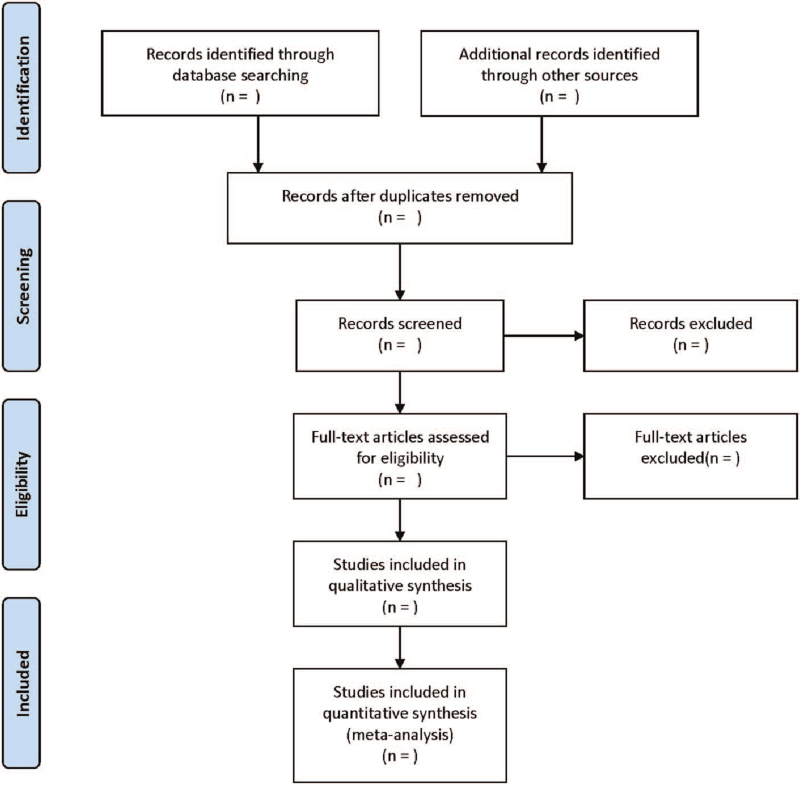
Flow diagram of study selection process.

Literature screening and data extraction will be independently conducted by two researchers and cross-checked. Any disagreement will be solved by the third researcher after discussion. The following data will be collected from each literature: First author, publication year, sample size, sex, age, course of disease, intervention measures, course of treatment, and outcome indicators. The screening flow chart of this study was demonstrated in Figure 1.

#### 2.6.2 Assessment of evidence quality

The Cochrane System Evaluation Manual version 5.1.0 bias risk assessment tool will be used to assess the quality of the included RCTs.^[23]^ The following contents will be assessed: whether the correct random method is reported; whether the allocation is hidden; whether the blind method is used; whether the object of withdrawal and loss of follow-up are explained; whether there are selective reporting results; whether there are biases from other sources. The assessment results will be categorized into low risk, high risk, or unclear. Two researchers will be responsible for checking the assessment results, and any disagreement will be solved by the third researcher after discussion.

The A MeaSurement Tool to Assess systematic Reviews 2 (AMSTAR-2) and Preferred Reporting Items for Systematic Review and Meta-analysis 2020 (PRISMA-2020) will be used by 2 independent reviewers to assess the methodological and reporting qualities of each included meta-analysis, respectively.^[24,25]^ The PRISMA tool is used to evaluate reporting quality, the statement of which contains a 27-item checklist and e ach item requires the reviewer to answer “yes,” “no,” and “partial yes.” Both AMSTAR-2 and PRISMA can be expressed as a percentage of items that meet “yes.”

The Grading of Recommendations Assessment, Development, and Evaluation tool is used to grade the quality of evidence of the main outcomes.^[26]^ Evidence may be reduced for various reasons, including study limitations, inconsistent results, indirect evidence, imprecision, or reporting bias. The quality of evidence can be classified into 4 levels: high (no degradation), moderate (1 degradation), low (2 degradations), and very low quality (≥degradations).

#### 2.6.3 Measures of therapeutic efficacy

Standard mean difference and 95% confidential interval will be pooled.

#### 2.6.4 Management of missing data

Missing data will be requested by Email; otherwise, the data will be excluded from the study.

#### 2.6.5 Assessment of heterogeneity and data synthesis

Methodological and reporting qualities of included systematic reviews will be presented as numbers and percentages, and the evidence mapping method will be used to visualize the results.^[27]^ Stata14.0 software will be used to draw an evidence network map to depict the comparison of the nonpharmacological interventions for each outcome indicator. *χ*^2^ Test will be performed to measure the heterogeneity among the direct comparison results, and *I*^*2*^ will be used to measure the heterogeneity. If there is no heterogeneity (*I*^*2*^ < 50%, *P* > .1), a fixed-effects model will be used for meta-analysis; Otherwise, a random-effects model will be adopted. Meanwhile, GEMTC 0.14.3 software will be used to perform mesh meta-analysis based on the Markov Chain-Monte Carlo (MCMC) fitting consistent model under the Bayesian framework. Four chains will be used for simulation, and the number of iterations will set at 50,000 (the first 20,000 for annealing and the last 30,000 for sampling). The estimation and inference will be carried out under the assumption that MCMC achieves a stable convergence state. The stability and consistency of the results will be evaluated by adopting the MCMC fitted inconsistency model.

#### 2.6.6 Assessment of reporting biases

“Comparison-adjusted” funnel plots will be depicted to evaluate publication bias.

#### 2.6.7 Subgroup analysis

Subgroup analysis would be applied based on the course of treatment and types of scales.

#### 2.6.8 Sensitivity analysis

The sensitivity analysis will be performed to test the stability of the results of meta-analysis.

#### 2.6.9 Ethics and dissemination

The content of this article does not involve moral approval or ethical review and would be presented in print or at relevant conferences.

## 3 Discussion

CRF is the most common symptom of a lung cancer that significantly influences the quality of life and prognosis of affected patients.^[28–30]^ Therefore, early prevention, diagnosis, and effective interventions for CRF are important. Although a large number of meta-analyses of nonpharmacological interventions of CRF in lung cancer patients have been published in peer-reviewed journals, their reporting and methodological qualities remain unclear and conflicting. Therefore, the present study can address the above issues through a network meta-analysis.

This study has several strengths. First of all, this is the first overview of a meta-analysis of nonpharmacological interventions of CRF in lung cancer patients. In addition, we will use the PRISMA-2020 and -2 tools to evaluate the reporting and methodological qualities of the identified meta-analyses, and the assessment results will be visualized using the evidence mapping approach. Secondly the network meta-analysis enables aggregation and comparison of all available treatments reported in studies involving in the identified meta-analyses. Ranking results of these interventions will be conductive to establish clinical practice guidelines for clinical decision making.

## Author contributions

**Conceptualization:** Lingyan Zhao, Jia Zeng.

**Data curation:** Lingyan Zhao and Ping Shi.

**Formal analysis:** Ping Shi.

**Funding acquisition:** Lingyan Zhao.

**Investigation:** Ping Shi, Xiaomin Xiong.

**Methodology:** Xiaomin Xiong.

**Project administration:** Jia Zeng, Lingyan Zhao.

**Resources:** Xiaomin Xiong.

**Software:** Xiaomin Xiong, Jia Zeng.

**Supervision:** Lingyan Zhao.

**Validation:** Lingyan Zhao.

**Visualization and software:** Lingyan Zhao.

**Visualization:** Jia Zeng.

**Writing – original draft:** Lingyan Zhao and Jia Zeng.

**Writing – review & editing:** Lingyan Zhao and Jia Zeng.
